# Growing Green Roofs, City by City

**DOI:** 10.1289/ehp.115-a306

**Published:** 2007-06

**Authors:** David A. Taylor

Green roofs—rooftops that are partially or completely covered with vegetation growing in soil medium over a waterproof membrane—have gained momentum over the past six years as building owners recognize their advantages over conventional roofing in terms of better energy efficiency and reduced rain runoff. Now local governments are exploring incentives for moving the practice into the mainstream. A look at cities that are leading the country in green roof coverage reveals a growing range of policy tools.

## Capital Growth

Alexi Boado, low-impact development coordinator for Washington, DC’s District Department of the Environment (DDOE), says the city began seriously examining green roofs for stormwater control five years ago, when the DC Water and Sewer Authority provided $300,000 for green roof development as part of a court-ordered settlement. Those funds, managed by the nonprofit Chesapeake Bay Foundation, seeded a program of incentive grants that encouraged eight builders to choose green roofs over other traditional devices as their primary stormwater control device (stormwater control plans are required for any new construction or redevelopment of more than 5,000 square feet in the District). Builders also have a procedural incentive: designs that include a green roof in the stormwater control plan receive expedited processing.

To build local engineering design and green construction capacity and catalyze interest in green roofs, the DDOE is working with the USDA Natural Resources Conservation Service to offer almost $800,000 in complete design-and-build services for select public and commercial properties. This program is slated to begin in the summer of 2007. In addition, as part of a cash grants program, DDOE and its sister agencies are in the process of installing green roofs on three new community recreation centers, two public schools, and one housing development. Previous grants have subsidized some of the first green roofs in the District, as well as the implementation of many other innovative stormwater control practices such as rain gardens and permeable surfaces. The District allotted about $500,000 in 2007 to innovative stormwater control grants in addition to the Natural Resources Conservation Service partnership.

Dawn Gifford, program coordinator of the nonprofit DC Greenworks, has seen a shift in green roof installations from mainly commercial buildings to a mix of commercial and residential. DC Greenworks has dedicated itself to installing green roofs throughout the city; a high-profile demo model they installed at 1425 K Street NW in 2004 has drawn more than 3,000 visitors and inspired similar projects across the metropolitan area. Doug Siglin, director of federal affairs for the Chesapeake Bay Foundation, explains the public policy perspective behind the interest in green roofs: one problem in the Anacostia River, which runs through Washington, DC, and in the bay generally, is too much erosion, with silt increasing water turbidity. Most erosion comes from stormwater runoff; green roofs help moderate that blast of runoff from precipitation events, and therefore help local governments deal with rainwater by detaining, retaining, and absorbing it where it first hits.

Chicago officials see another public health benefit in moderating the city’s “heat island” effect (defined as urban and suburban areas having temperatures up to 10°F higher than nearby rural sites). Heat islands spike energy demands, air pollution levels, and heat-related illnesses such as heat exhaustion and heat stroke. With climate change, says Sadhu Johnston, the city’s commissioner for the environment, Chicago can expect hotter and drier summers—conditions that the heat island effect will only exacerbate.

Johnston says green roofs can help avert heat wave–related deaths, citing studies that show lower temperatures on green roofs compared with traditional roofs, and reduced air-conditioning use in buildings with green roofs. According to the 2004 *Green Roof Test Plot 2003 End of Year Project Summary Report* by environmental engineering firm MWH, which is posted on the City of Chicago website, the mean temperature of green roof areas in the heat of the day (between 12:30 and 4:30 pm) was up to 31% cooler than other roof types.

## Salad Days of Incentives

Chicago mayor Richard Daley, Jr., installed a green roof on City Hall after returning from a 1999 visit to Europe, where he saw one in action. “That [installation] really sparked people’s imagination,” says Johnston. The city also offered grants and stormwater credits (a reduction in city fees for stormwater management) to prospective green roof owners to jumpstart the practice. Today Chicago leads the country in green roofs, with 300 buildings comprising some 3 million square feet of green roofing, says Johnston. Most such roofs are on commercial buildings (including Target and McDonald’s) but many are on civic buildings and smaller stores.

Incentives also evolved in Portland, Oregon. Tom Liptan, an environmental specialist with the city Bureau of Environmental Services, says about 20 years ago the city added a floor area ratio (FAR) bonus to its building code whereby builders could get permission to build extra square footage (either up or out) by employing favored practices. In the 1990s Liptan realized that European-style green roofs might help Portland with stormwater control. He put a green roof on his garage in 1996 and measured rain runoff for two years. Eventually, the city adjusted its FAR bonus to include green roofs as a favored practice. One builder who installed 4,000 square feet of green roof, for example, received permission to build an extra 12,000 square feet of building density; the builder was able to add six condo units, then selling for $395,000 each. “They spent sixty thousand dollars to get two million dollars’ worth of additional sellable property,” says Liptan.

Chicago likewise gives a density bonus for green roofs in its central business district, which permits developers to increase the number of units allowed on a piece of property. The city also offers an “express lane” for the permit process. Johnston says with a green roof in the design, “you get a dedicated team of reviewers, and you get a permit in thirty days” as compared to the typical 90 to 100 days. Plus, the city waives the developer’s fee for processing the building permit application.

Inducements include sticks as well as carrots. Chicago requires any developer who receives city assistance (for example, to rehabilitate a brownfield) to include a green roof. Builders have reservations about that approach, since green roofs have a higher initial cost. Stuart Match Suna, cofounder of production company Silvercup Studios, which installed a green roof on its building in Long Island City, New York, is leery of regulatory mandates. “I would be reluctant to require them,” he told the September 2006 issue *Metropolis* magazine. “That would make New York City that much more expensive [to rent or own property in].” Mary Margaret Hiller, marketing and communications director for Washington, DC–based developer Akridge, adds, “There is a premium to pay for a green roof, so I think it’s up to the developer whether they feel a green roof is necessary.”

But builders also note growing client interest. “If you want to be a player, you have to be up on these technologies,” says Hiller. Akridge, for example, has gone from having no green roof designs several years ago to managing three green-roofed properties now and developing designs for three more, including one property that will be completed next year.

## Tools for the Trade

After years of clarifying green roof practices and benefits for builders, nonprofit groups and associations are helping governments explore the economics and policies affecting the technology, sometimes with industry funding. In New York City, Earth Pledge, an industry association of green builders, has worked with city officials to oversee design and construction of seven roof projects on condos and apartment buildings in the Bronx, Brooklyn, and Harlem, according to executive director Leslie Hoffman. “There is recognition that multifamily residential is a very interesting opportunity for green roofs,” she says, estimating that close to half of Earth Pledge’s green roof projects are on apartment buildings.

In February 2007, Earth Pledge and the nonprofit Green Roofs for Healthy Cities received a $300,000 grant from the Home Depot Foundation to foster green infrastructure in Minneapolis, Los Angeles, Atlanta, and other cities. “We’re focused on developing smart tools [for policy makers and developers],” says Hoffman. These include models that show planners how much water a green roof at a given location is likely to capture, and GIS-based models that show how a larger-scale shift to green roofs would affect stormwater outflow at the watershed level.

There is no national inventory of green roof policies, but in April 2007 Green Roofs for Healthy Cities launched the Green Roofs Tree of Knowledge, a database on research and policy related to green roof infrastructure. At a regional level, in March 2007 the Washington, DC–based nonprofit group RESOLV prepared a report, *Public Funding Incentives for Private Residential and Commercial Watershed Protection Projects*, for officials in Montgomery County, Maryland. The report reviewed the region’s rules and incentives, highlighting case studies nationwide, with the aim of improving watershed health. The report summary stresses targeting priority sub-watershed areas, voluntary action by property owners, and public education. Boado says the EPA is doing a similar study.

## Raising the Roof

For everyone, the state of green roof implementation is in a learning phase. “Most of the solutions we’ve come up with are home-grown,” Johnston says of Chicago’s policies. “Most we haven’t seen used in this country before.”

The EPA cites green roofs as one option for ameliorating the heat island effect. Hoffman suggests that the EPA could incorporate green roofs into incentives for cities to comply with the Clean Water Act. For example, by developing a green roof plan, a city might gain a postponement against federal compliance requirements. Such an option would likely be seen as an opportunity rather than a regulatory burden.

This would keep the push for green roofs at the city level, where rivalries keep advancements bubbling. “Chicago is the leading competition for us—friendly competition,” Portland’s Liptan says. Washington’s Boado confirms this sporting element. “Chicago has thrown down the gauntlet,” he says. “We’re the nation’s capital, and we want to be the greenest city in America.”

Siglin says that relatively small subsidies can nudge developers. “It’s a good policy lesson for governments,” he says: with a few grants and educational outreach, governments can foster a practice that reduces the public costs of managing runoff and water pollution abatement. As a policy tool, then, green roofs show unexpected potential. “That,” says Siglin, “helps the taxpayer in many ways.”

## Figures and Tables

**Figure:**
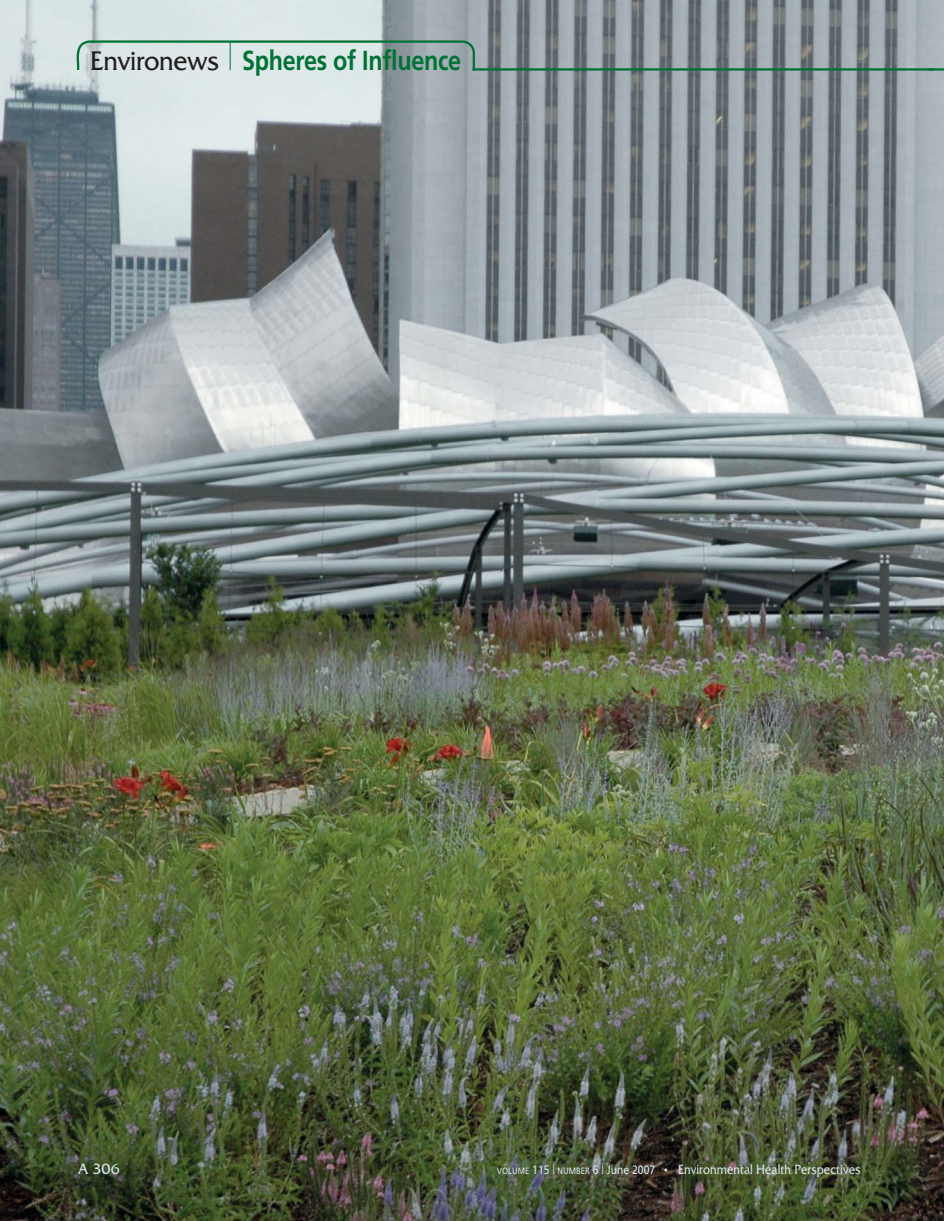
Millennium Park atop Chicago’s City Hall covers 24.5 acres. The public park includes numerous fountains, sculptures, and botanical garden spaces, as well as performance facilities, restaurants, and a skating rink.

**Figure:**
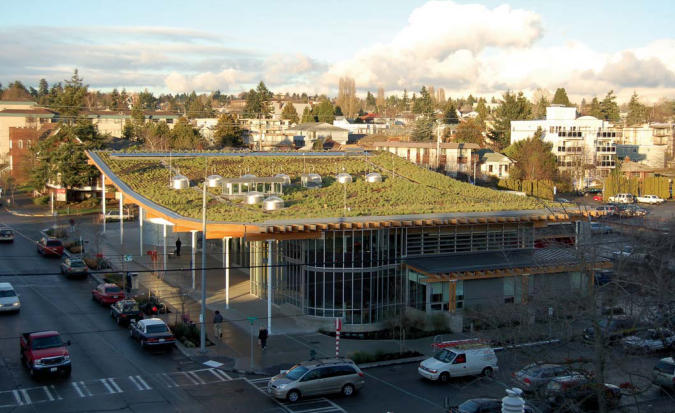
The Ballard Library in Seattle incorporates solar panels into its green roof design. Energy generated from these panels is fed back in to the city’s power grid. The curved roof create six microclimate conditions, each a separate exposure with differing water retention properties, based on slope and orientation.

**Figure:**
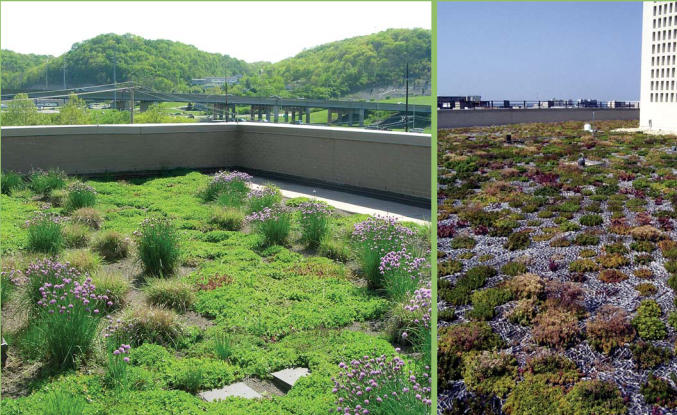
(left) Stormwater flows from the green roof at Sanitation District No. 1 in Fort Wright, Kentucky, into a naturalized wetland, then a retention basin, a detention basin, step pools, and finally into Banklick Creek. (right) One of Washington, DC’s first green roofs was installed at 1425 K Street NW.

**Figure:**
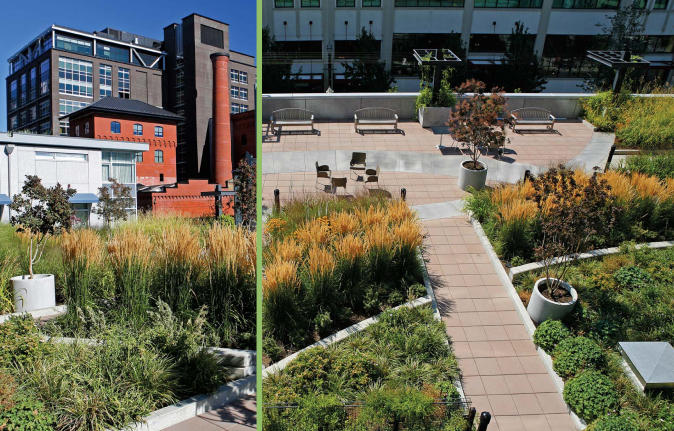
Two views of the Louisa, a Portland, Oregon, residential high-rise with 242 apartments and ground-floor retail. Other green features of the building include high-efficiency glazing, low-toxicity building materials and finishes, and locally sourced construction materials.

**Figure:**
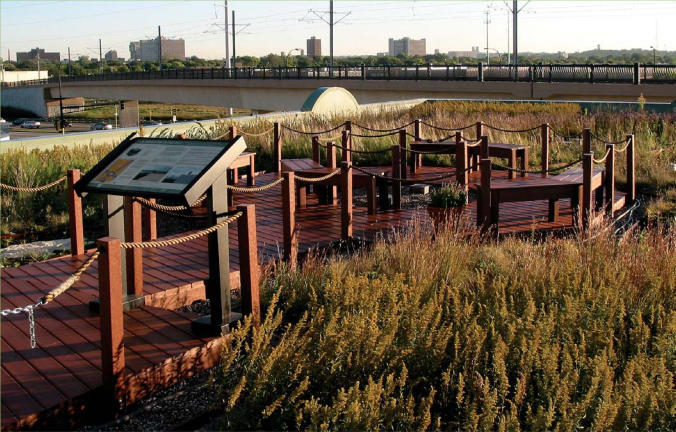
The green roof on the Phillips Eco-Enterprise Center in Minneapolis, stocked with native plants, educates visitors about the diminishing local bedrock bluff prairie ecosystem. The roof also offers a pleasing view to passengers on the nearby elevated train.

